# The effect of contraceptive access reform on privately insured patients: Evidence from Delaware Contraceptive Access Now

**DOI:** 10.1371/journal.pone.0280588

**Published:** 2023-01-23

**Authors:** Maranna Yoder, Michel Boudreaux

**Affiliations:** 1 Department of Economics, University of Maryland, College Park, Maryland, United States of America; 2 Department of Health Policy and Management, University of Maryland, College Park, Maryland, United States of America; The University of Texas MD Anderson Cancer Center, UNITED STATES

## Abstract

**Background:**

Many states are implementing comprehensive programs aimed at reducing persistent barriers to contraceptive care. Evidence on the effectiveness of these programs is essential for practice improvement and policy development.

**Objective:**

To evaluate changes in the probability of initiating a contraceptive method by women with employer sponsored insurance after implementation of Delaware Contraceptive Access Now (DelCAN), a statewide initiative that aimed to increase access to long-acting reversible contraceptives (LARCs).

**Design, setting, and participants:**

We used a difference-in-differences design to examine contraceptive initiation rates. Data came from IBM Marketscan and covered women age 15–44 enrolled in employer sponsored insurance. The primary outcome was insertion of a LARC, both in the overall study population and in the immediate postpartum (IPP) setting. Secondary analysis examined changes to other contraceptive method types.

**Results:**

The cohort of 4,550,459 enrollees generated a sample of 11,888,837 person-years and 615,670 childbirth hospitalizations. Difference-in-differences estimates suggested that DelCAN was associated with a 0.3 percentage point (95% CI [0.2, 0.5], p<0.001) increase in the LARC insertion rate in the overall study population and a 0.4 percentage point increase (95% CI [0.2, 0.6], p<0.001) in the percent of births adopting IPP LARC. Associations between DelCAN and LARC insertion appeared stronger for adolescents compared to older women. Results for other method types were less consistent.

**Conclusions:**

A comprehensive statewide program was associated with increased LARC insertion rates among enrollees with employer sponsored insurance. Understanding the effect of these programs is critical for on-going policy development for states engaged in contraceptive access reform.

## Introduction

About half of all pregnancies in the United States occur earlier than desired or are not explicitly wanted [1]. Unintended birth can have a detrimental impact on the health and economic wellbeing of pregnant people and their families [2,3]. Providing access to affordable and effective contraception is a prominent policy strategy in the US for reducing the risk of unintended pregnancy [4]. However, financial and logistical barriers have hindered access to the most effective methods, including long-acting reversible contraceptives (LARCs) [5–7].

Over the past decade, the states have undertaken a number of reforms meant to improve access to contraceptives. These reforms have typically focused on, but not been limited to, Medicaid payment reform. An especially popular reform has been to carve-out reimbursement for LARCs delivered in the hospital, immediately after delivery [8–13]. While 43 states have adopted some version of this reform [14], existing evidence on its effects is thin and mixed. Evidence from South Carolina suggests greater aggregate adoption of immediate postpartum (IPP) LARC [11], while evidence from Wisconsin suggests no meaningful increase in take-up of IPP LARC post-reform [10], though both settings suggest take-up rates vary significantly across medical providers.

A handful of states, including Colorado, Delaware, Massachusetts, South Carolina, Illinois, and others, have taken a comprehensive approach to contraceptive access reform by implementing coordinated programs that are designed to improve access not only for postpartum Medicaid patients, but for all patients that desire a contraceptive, at any reproductive stage [15,16]. These programs typically include some combination of payment reform, clinical training, and public awareness campaigns. While these coordinated statewide programs are growing in popularity, much remains unknown about how effective they are at improving contraceptive access.

While the components of these programs tend focus on publicly supported patients, they have the potential to impact patients covered by private payers through provider training and public awareness campaigns. It is also possible that payment reform in the public market could have spillovers on private patients through the behavior of providers.

This study estimated the association of Delaware Contraceptive Access Now (DelCAN) on LARC insertion among patients with employer-sponsored insurance. We consider changes in overall LARC insertion rates and in IPP LARC. DelCAN is a statewide program focused on improving access to contraceptives, and LARCs in particular [17]. The program began in 2015 and included a coordinated bundle of Medicaid payment reforms, clinical training on patient-centered contraceptive counseling and LARC provision, and business operations training focused on revenue cycle management. Training occurred at all Title X clinics, the largest private outpatient clinics, and five out of the six maternity hospitals in Delaware. The new Medicaid payment reforms allowed for reimbursement of LARC devices placed immediately postpartum, separately from the bundled in the global payment and delivery fee, and a carve-out for separate reimbursement of LARC devices from the standard Federal Qualified Health Center visit rate. A public awareness campaign that advertised the availability of free contraceptives and the location of high capacity providers was fielded in 2017 and was successful at increasing attendance at reproductive health clinics [18]. More details on the program are provided elsewhere [17].

Understanding the association of DelCAN, and similar comprehensive statewide programs, in the privately insured population is particularly important given that 63% of women age 19–44 are covered by private insurance and 44% of unintended births are to privately insured people [19–21]. Because the privately insured can access contraceptives without any out-of-pocket expense via the Affordable Care Act’s contraceptive mandate, the primary mechanisms through which DelCAN might be associated with LARC insertion are improvements in clinical practice and increases in patient awareness about the availability of affordable services. Additionally, DelCAN’s Medicaid payment reform and related provider training may have effects on providers’ capital resources or practice patterns that affects provision of LARCs for patients with employer sponsored insurance.

## Materials and methods

### Data and study sample

We used the IBM Marketscan Commercial Claims and Encounters Database of private health insurance claims to measure initiation of prescription contraceptives among enrollees in employer-sponsored insurance [22]. This data source contains enrollment information, inpatient claims, outpatient claims, and pharmacy claims for enrollees. Our study sample was comprised of enrollees identified as women age 15–44, and includes both policyholders and dependents. The sample period was 2012–2019 and we included all enrollees in employer sponsored insurance in the Marketscan database that resided in Delaware. Due to budget constraints, we obtained data for a large random sample of eligible individuals in other states as our primary control group. The sampling was done at the enrollee level and was carried out by the data provider. We excluded individuals from Colorado, Iowa, Massachusetts, South Carolina, Texas, and Washington due to contemporaneous family planning initiatives (or contractions) in those states. We explore alternative control groups in a sensitivity analysis in the Supporting Information.

We analyzed two analytic samples. The first sample is structured at the person-year level and is used to analyze changes in the overall LARC insertion rate. This sample captures LARC insertions that occur in both inpatient (e.g. IPP) and outpatient settings that might include care received at reproductive health clinics, in primary care, or at an OB-GYN visit. For this sample, we followed Office of Population Affairs (OPA) guidance and excluded enrollees who had an indication of infertility or pregnancy during the year and enrollees who recorded a live birth in the last two months of the calendar year [23]. These restrictions were to ensure all enrollees in the sample had a minimum amount of time at risk for contraceptive initiation. This analysis sample includes both pre-conception patients and postpartum patients who delivered prior to November of each year.

While this data source can identify the same individuals over time, we do not require that individuals are present in every year of the sample. Doing so would reduce the power of the study, as less than 5% of individuals are observed in all years. Additionally, requiring a panel structure imposes selection on the participants. Marketscan is derived from data at large employers, so one must remain enrolled with a Marketscan employer and meet the OPA criteria for all eight years to be included in a panel (e.g. ruling out transitions to or from parental or spousal insurance, job changes, employer disengagement with Marketscan, and pregnancy). In the Supporting Information we examine how sample composition changes over time. Finally, individuals are included in the sample period in years after they have a LARC insertion, provided they meet other sample criteria, to be consistent with individuals who enter the sample with a LARC, whom we are unable to identify and exclude. Our estimates can best be interpreted as a lower bound of the effect of DelCAN among individuals at risk of LARC insertion.

The second analysis sample is used to study changes in IPP LARC provision. This sample is structured at the hospitalization level and includes admission and discharge dates for each hospitalization. We subset the data to hospitalizations during which a live birth is recorded. Because this sample is used to study LARC insertion during hospitalization, we use the full set of hospitalizations occurring at any point during the year.

### Outcomes

We focus on LARC insertion because most program activities centered around the unique barriers to LARC access [17]. We identify claims for contraceptive methods using medical and drug billing codes (ICD-9, ICD-10, HCPCS, CPT, and NDC codes) compiled by OPA [23]. These codes identified claims for LARC methods (intrauterine devices and contraceptive implants) and other contraceptive methods. LARC insertion, rather than use, is both relevant to policy and has practical measurement advantages. Because LARCs are long lasting and require few follow-up appointments, on-going LARC use is impossible to accurately measure in claims data. As a result, we focus on LARC insertion as the outcome of interest. For the person-year sample, we consider an individual as having a LARC insertion if they record a claim with one of the identified medical or drug billing codes at any point during the year. For the postpartum sample, we consider an individual as having an IPP LARC insertion if they record a claim for LARC insertion during the dates of hospitalization for childbirth. The LARC insertions recorded in the person-year sample are inclusive of the IPP LARCs identified in the postpartum sample for patients who gave birth prior to November.

In a secondary analysis, we also analyzed initiation of other contraceptive methods such as moderately effective contraception methods (pill, patch, ring, shot, diaphragm), female sterilization procedures, and any prescription method. Further detail on the data construction and analysis of these contraceptive methods is provided in the Supporting Information.

### Demographic and population characteristics

We used the Marketscan data, the American Community Survey (ACS), and the Area Health Resource File (AHRF) to measure a set of individual and state-by-year covariates [24,25]. At the individual level, we obtained age, an indicator for living in an urban area, an indicator for being the policyholder, and an indicator for whether the insurance plan was a high deductible health plan, as recorded in Marketscan. From the ACS, we obtained the shares of the state-year population with private health insurance and public health insurance, the racial composition of women age 15–44 with employer sponsored insurance, and the median household income in the state. From the AHRF, we use the percentage of women of reproductive age in the state living in a county without a maternity hospital, the number of primary care doctors and OBGYNs per 10,000 residents, and the number of rural health centers or federally qualified health centers per 100,000 residents. Finally, we included indicators by state and year that identify if state law required insurance coverage of over-the-counter contraceptives or male vasectomy, and if state law allowed an extended supply of birth control pills to be prescribed or allowed pharmacists to prescribe contraceptives.

### Statistical analysis

We used a difference-in-differences design that compared changes in the outcomes in Delaware, from before (2012–2014) to after program implementation (2015–2019), to changes in the control states. The design assumed that the changes observed in the control states represented what would have happened in Delaware had the program not been implemented. We implemented the design in two ways. First, changes from the pre to the post periods were compared, as described above. Then, an event study approach was used to compare changes in outcomes for each year relative to the difference in 2014 (the last pre period year). The event study model provided evidence on whether outcomes were changing differently in Delaware relative to control states before the program was implemented, which would reduce confidence in the study design. It also provides evidence if post period effects changed over time as program components were rolled out and patients learned about the program.

To conduct the difference-in-differences, linear probability models were used where the outcome of interest was an indicator whether the individual initiated a particular type of contraception during the calendar year for the person-year sample or during hospitalization for the postpartum sample. Standard errors were clustered at the individual level. While we considered clustering at the state level which is common in the difference-in-differences literature, in our setting state-level clustering is not appropriate because there is only a single treated cluster and standard methods for estimating standard errors will substantially understate sampling variance [26,27]. All regressions included state fixed effects to account for unique factors about states that remained constant over time and year fixed effects that accounted for unique factors about years that were the same across states. The coefficient of interest was the interaction term between the Delaware indicator and the post indicator (or the relative time indicators in the event study model). We report results with and without controlling for the covariates described above.

Analyses were conducted for all eligible enrollees and for subgroups of enrollees defined by age (15–17, 18–29, 30–44) and whether the enrollee was the policyholder or a dependent (either spouse or child of the policyholder). The policyholder/dependent subgroups are important because a dependent’s use of services is not confidential from the policyholder. Dependents may have responded differently to the availability of new contraceptive services as a result.

Analyses were conducted in SAS version 9.4, R version 4, and Stata MP Version 17.0.

## Results

Our person-year sample included 223,913 person-years (71,875 unique individuals) in Delaware and 11,664,924 person-years in the control states (4,478,584 unique individuals). The postpartum sample included 11,133 hospitalizations for childbirth in Delaware and 604,537 in the control states. Table 1 presents summary statistics from both our samples across the entire 2012–2019 time period, for Delaware and the control states respectively. Due to the large sample size most comparisons are statistically significant even though most differences are small and not meaningful, as illustrated by standardized differences that are less than 0.1 [28]. Exceptions to note are that enrollees in the comparison states are more than twice as likely to be in a high deductible plan (8.1% vs. 3.0%) and enrollees in Delaware are more likely to live in urban areas (95.9% vs. 87.7%).

**Table 1:** Summary statistics for women age 15–44 in delaware and control states, 2012–2019.

Variable	Delaware	Control	Standardized Difference	p-value
**Person-Year Sample**				
Age	29.6	29.7	-0.02	0.000
Age 15–17 (%)	9.9	9.4	0.01	0.000
Age 18–29 (%)	39.3	39.0	0.01	0.000
Age 30–44 (%)	50.8	51.6	-0.01	0.000
In urban area (%)	95.9	87.7	0.30	0.000
Policyholder (%)	48.6	44.7	0.08	0.000
High deductible health plan (%)	3.0	8.4	-0.24	0.000
Postpartum (%)	4.4	4.5	-0.01	0.000
N (person-years)	223,913	11,664,924		
**Postpartum Sample**				
Age	30.3	30.4	-0.02	0.100
Age 15–29 (%)	41.2	41.5	-0.01	0.600
Age 30–44 (%)	58.8	58.5	0.01	0.600
In urban area (%)	95.6	87.8	0.29	0.000
Policyholder (%)	56.2	48.7	0.15	0.000
High deductible health plan (%)	2.4	8.5	-0.27	0.000
N (births)	11,133	604,537		

Source: IBM Marketscan Commercial Claims and Encounters Database (2012–2019). Notes: Observations for all LARC insertions are at the “person-year” level and observations for IPP LARC are at the birth level. “Percent in urban area” refers to the percentage of person-year observations where the enrollee was located in a Metropolitan Statistical Area. The p-value reported is for a t-test of whether the means for Delaware and the control states are equal.

**Fig 1:**
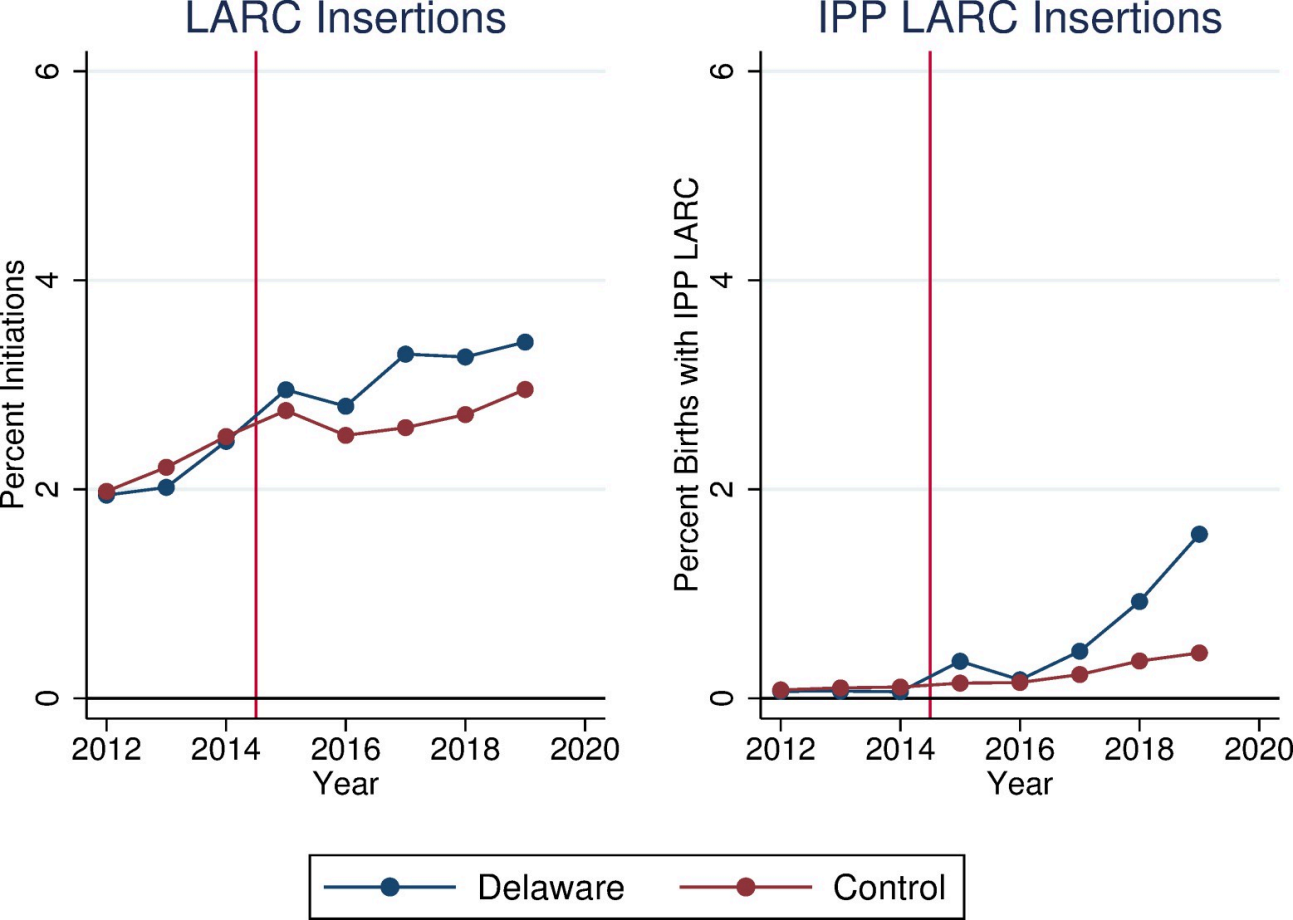
Average LARC insertion rates and IPP LARC insertion rates among women age 15–44 in delaware and control states. Source: IBM Marketscan Commercial Claims and Encounters Database (2012–2019). Notes: The rate reported in the left-hand panel each year is calculated as the number of women having a LARC device inserted in that year, divided by the total number of women enrolled in a plan in the Marketscan database. The rate reported in the right-hand panel is calculated as the number of births in a hospital where a LARC device was provided during the dates of hospitalization, divided by the total number of births occurring in a hospital recorded in the Marketscan database for each year.

Fig 1 shows LARC insertion rates by year. Prior to program implementation in 2015, Delaware had slightly lower rates of LARC insertion than control states. However, in the post period, Delaware’s LARC insertion rate surpassed that of control states for every year through 2019. Both Delaware and the control states had minimal rates of IPP LARC usage prior to program implementation, but Delaware’s IPP LARC rate was greater than that of control states in every year after 2015, with particularly large increases in 2018 and 2019.

Our main results are presented in Table 2. Among enrollees age 15–44, the average unadjusted probability of receiving a LARC in control states increased from 3.1 percent on average in 2012–2014 to 4.0 percent in 2015–2019, but changed from 3.0 percent to 4.3 percent in Delaware. Our adjusted difference-in-differences estimates suggest that DelCAN was associated with an increase in the probability of a LARC insertion by 0.3 percentage points in an average post-period implementation year (95% CI: 0.2, 0.5; P<0.001). This represents a 11% change in LARC insertion from the Delaware baseline rate.

**Table 2:** Difference-in-differences estimates (percentage points) of the Effect of DelCAN on LARC insertion among 15–44 year olds enrolled in employer sponsored insurance.

	Unadjusted Insertion Rates				Difference-in-Differences	
	Delaware		Control			
	Pre	Post	Pre	Post	Unadjusted	Adjusted
All LARC Insertions	3.0	4.3	3.1	4.0	0.5*** [0.3, 0.6]	0.3*** [0.2, 0.5]
IPP LARC Insertions	0.1	0.6	0.1	0.2	0.4*** [0.2, 0.6]	0.4*** [0.2, 0.6]

Source: IBM Marketscan Commercial Claims and Encounters Database (2012–2019). Notes: Difference-in-differences estimates are in percentage points. Both the unadjusted and adjusted difference-in-difference estimates include state and year fixed effects. The adjusted model controls for individual level covariates and state by year covariates for demographics, health care access, and other state contraceptive policies. See the text for sample inclusion rules and full list of covariates. Statistical tests account for clustered standard errors at the individual level and 95% confidence intervals are shown. †p<0.1, *p<0.05; **p<0.01; ***p<0.001.

We also found that DelCAN was associated with an increase the rate of IPP LARC insertions (Table 2). In the control states, IPP LARC insertions increased from 0.1 percent of births in 2012–2014 to 0.2 percent of births from 2015–2019, but increased from 0.1 percent to 0.6 percent of births in Delaware. The adjusted difference-in-differences estimate is 0.4 percentage points (95% CI: 0.2, 0.6; P<0.001).

**Fig 2:**
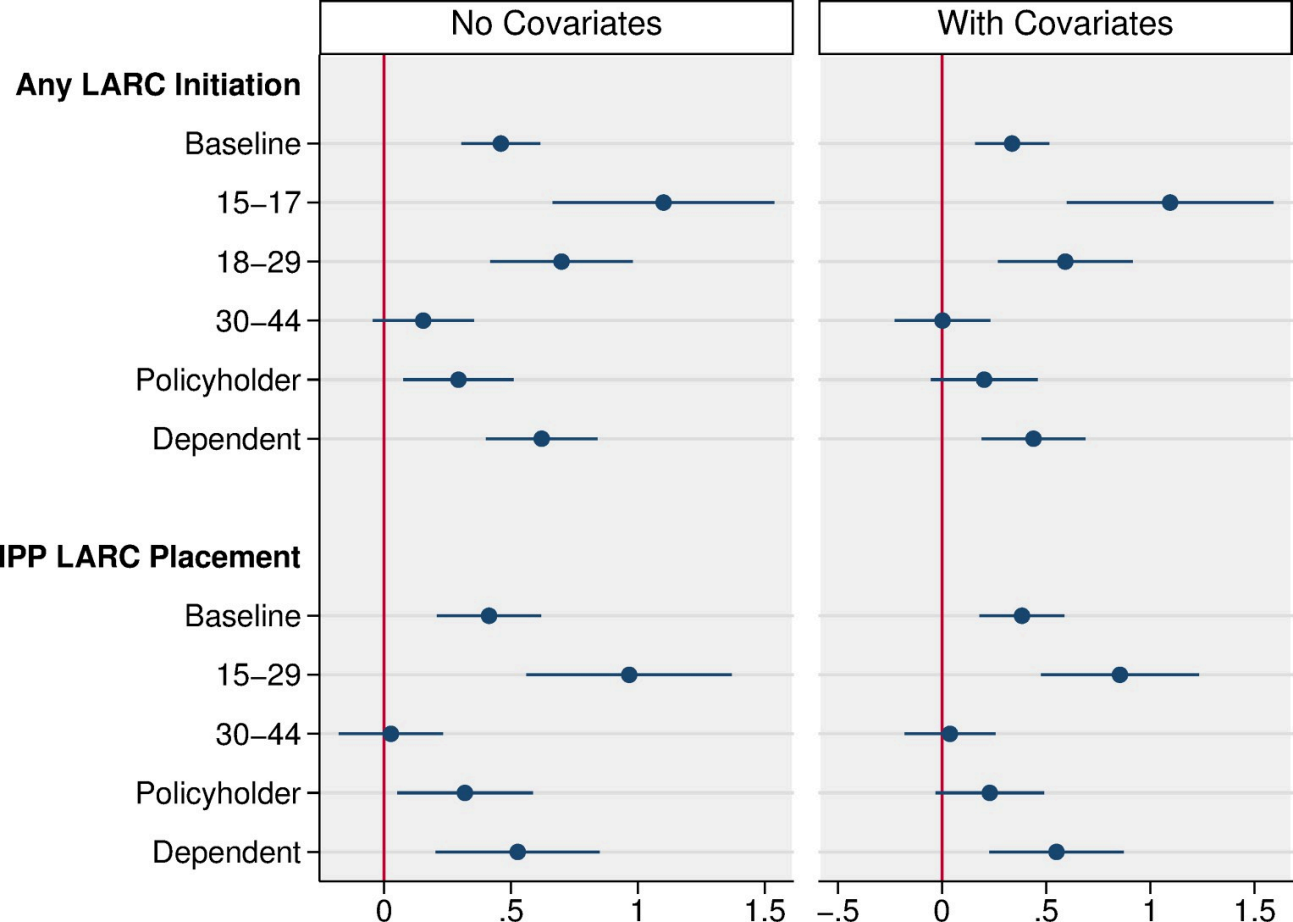
Difference-in-differences effect of the DelCAN program on LARC insertions, for women age 15–44 by age group and policyholder status. Source: IBM Marketscan Commercial Claims and Encounters Database (2012–2019). Notes: Difference-in-differences estimates are from a linear probability model estimated with individual data at the person-year level for any LARC insertions and at the birth level for IPP LARC placements. Both the unadjusted and adjusted difference-in-difference estimates include state and year fixed effects. The adjusted model controls for individual level covariates and state by year covariates for demographics, health care access, and other state contraceptive policies. See the text for sample inclusion rules and full list of covariates. Standard errors are clustered at the individual level and 95% confidence intervals are shown.

Fig 2 presents subgroup results. The program’s association with LARC insertions of 0.3 percentage points is driven most strongly by enrollees age 15–17, among whom the program was associated with a 1.1 percentage point (95% CI 0.6, 1.6; P < 0.001) increase in the probability of LARC insertion after the program, representing a 51% increase from the Delaware baseline rate of 2.2 percent. That is, among enrollees age 15–17, 2.2 percent of enrollee-years in Delaware in the pre-period reported a LARC insertion, and we estimate that DelCAN was associated with a 1.1 percentage point increase in the LARC insertion rate among this age group. For enrollees age 18–29, the program was associated with a 0.6 percentage point (95% CI 0.3, 0.9; P < 0.001) increase in the probability of LARC insertion after the program, a 16% increase from Delaware baseline at 3.7 percent. The data do not suggest an association for enrollees age 30–44 on the probability of receiving a LARC. We also find stronger associations for enrollees age 15–44 that are dependents, who are associated with a 0.4 percentage point (95% CI 0.2, 0.7; P < 0.001) increase in the probability of LARC insertion after the program compared to 0.2 percentage points (95% CI -0.1, 0.5; P = 0.12) for policyholders.

The subgroup patterns are mirrored in the patterns for insertion of IPP LARC. Younger enrollees age 15–29 account for the entirety of the effect and were associated with a 0.9 percentage point (95% CI 0.5, 1.2; P <0.001) increase in the probability of having an IPP LARC insertion after the program, compared to a statistically insignificant 0.04 percentage point increase (95% CI -0.2, 0.3; P = 0.73) for enrollees age 30–44. We cannot estimate separate effects on IPP LARC for enrollees age 15–17 due to the small number of births in our sample among this age group.

For all outcomes and subgroups, the adjusted and unadjusted models suggested similar results. This provides support for our study design because it suggests little meaningful differential compositional shifts and that our results are unlikely to be biased by unobserved confounding. It also suggests that the inclusion of covariates does not itself bias our estimates as recent methodological work suggests can happen [29]. We present additional evidence on baseline characteristics by age group in the Supporting Information. While adolescents are enrolled in high-deductible health plans and live in urban areas at a similar rate as adult enrollees, they are substantially less likely to be policyholders or be in a postpartum period in a given year. Both being a policyholder and being postpartum are positively associated with LARC insertion in the adult sample, so these differences in reported characteristics do little to explain the greater response observed among teenage enrollees.

Results from the event study models are presented in the Supporting Information. The event study models suggested no significant differential pre-period trending for overall LARC insertions and IPP LARC, which supports the validity of the study design. Additionally, S2 Table in S1 File presents the results of a test of differential pre-trends. We cannot reject the null hypothesis of parallel pre-period trends for either LARC outcome.

The event study models suggest that the effect on overall LARC insertions is greatest in the first through fourth years after DelCAN began implementation. The peak occurs in 2018, the fourth year after implementation began, with an estimated 0.5 percentage point increase (95% CI 0.1, 0.9; P = 0.01) in the probability of receiving a LARC for women in Delaware. For IPP LARC usage, the largest effects are in 2018 and 2019, with estimated effects of 0.7 (95% CI 0.1, 1.3; P = 0.02) percentage points for 2018 and 1.3 (95% CI 0.5, 2.1; P = 0.001) percentage points for 2019.

### Sensitivity analysis and secondary outcomes

We explored different control groups. Our preferred estimates (Table 2) are based on a broad control group that only excluded states if they enacted major reproductive health related reforms. However, many control group states did enact smaller reforms and our assumption was that Delaware would have behaved similarly to the average control group state in the absence of DelCAN. That decision was supported by pre-trends testing that did not suggest a violation of the parallel trends assumption. However, there is uncertainty about the best possible control group and consistent evidence across alternatives would provide confirmatory evidence. S3 Table in S1 File suggests that removing states that are relatively more active in contraceptive policymaking and/or only using neighboring states suggests the same conclusions as our preferred control group.

We also examined if the composition of the sample changed differently in Delaware than the control group which might suggest confounding bias. The largest differential change was the share of the sample living in an urban metropolitan area (S4 and S5 Tables in S1 File). In Delaware, the sample changed from 83.7% urban in 2012 to 100% urban by 2019. The control group changed from 85% to 90%. While such differential change is concerning, our main results are unchanged when we limit the analysis to patients living in urban areas (S6 Table in S1 File). Finding robust results in the urban sample in addition to our failure to reject parallel trends, similar results with and without covariate adjustment, and consistent results across alternative control groups suggests that our results are unlikely to be explained by compositional biases.

Finally, we attempted to measure if the program affected initiation of other contraceptive methods such as oral contraceptives. Further detail on data construction and results are provided in the Supporting Information. Results suggested no change to sterilization or any method initiation and small declines in moderately effective method initiation. However, the results were less conclusive than our analysis of LARC due to violations of the parallel trends assumption and challenges in measuring moderately effective method initiation.

## Discussion

We studied the association of a comprehensive contraceptive access program in Delaware with LARC insertion among people enrolled in employer sponsored insurance. Overall, our analysis suggests a 0.3 percentage point change in overall LARC insertions. That is, we estimate that DelCAN is associated with approximately an additional 3 privately insured women per 1,000 having a LARC insertion in a given year, and this increase represents about 11% of the pre-period rate of 30 women per 1,000. According to the ACS, there are approximately 115,000 women age 15–44 with employer sponsored insurance in Delaware in an average year during the sample period. Thus, in addition to DelCAN’s effects among Medicaid and Title X populations, we estimate DelCAN is associated with an additional 387 LARC insertions statewide per year among enrollees in private insurance (using unrounded estimates).

Associations were largest for teenage enrollees where the LARC insertion rate increased by 1.1 percentage points, or 56% from the baseline rate in Delaware. This result is consistent with previous work on DelCAN in the Medicaid program which found larger effects for adolescents than adults and with evidence from Colorado that found that a similar program was particularly salient for teenagers [30,31]. Larger effects for adolescents could reflect higher demand for LARC in that age group, especially in the context of lower baseline insertion rates relative to older adults, coupled with the broad scope of the training program, which included pediatric and other primary care providers [32]. Additionally, the DelCAN media campaign may have been more effective at reaching adolescents through online advertisements like social media and streaming ads [18].

Young adult enrollees also increased LARC insertion, but by smaller margins, and we did not find any evidence of increased LARC insertion for 30–44 year olds. We found stronger associations for dependents compared to policyholders suggesting that the lack of confidentiality that dependents encounter did not prevent them from taking advantage of new services.

We found a 0.4 percentage point change in IPP LARC insertion rates, which is a large increase from the baseline rate in Delaware. All of the IPP effect was concentrated among enrollees age 15–29, who experienced a 0.9 percentage point increase in IPP LARC insertion rates. We did not find evidence for increased IPP LARC usage among 30–44 year olds.

Our estimated effects on IPP LARC are broadly consistent with previous evaluation work in Delaware that found that the program was associated with a 30% increase in postpartum LARC use among privately insured patients, as measured in the Pregnancy Risk Assessment Monitoring System (PRAMS) [33]. However, our estimates are distinct from this previous study in that we directly measured hospital-based IPP, whereas the PRAMS measures postpartum contraceptive use up to several months after delivery in all care settings. Our IPP results are also consistent with results from a study of one medical center in Missouri that found that a similar IPP reform was associated with a 3 percentage point increase in the fraction of commercially insured births using IPP LARC one year later [13]. Like our setting, this intervention included both the Medicaid payment reform and institutional training and preparation. However, our results leverage a larger sample size and estimate the net effect among a heterogeneous statewide population of medical providers.

To our knowledge, this study represents the first population based estimates of a comprehensive statewide contraceptive access program on contraceptive patterns in the employer sponsored insurance market. Given the size of the privately insured population and the fact that privately insured patients often face the same supply side barriers to LARC that publicly supported patients do, understanding the experience of the privately insured is of importance to practitioners and policymakers [34,35].

Comparing effects observed among publicly supported patients to effects observed among the privately insured also provides some insights into the relative importance of payment reform (which only directly affected Medicaid) versus provider training. The results presented here are smaller than those reported by Boudreaux et al. (2020), who found a 3.2 percentage point change in LARC use among Title X patients (a 40% change) [36]. However, our estimates are not perfectly comparable to Boudreaux et al. (2020) because we studied LARC insertion and not use and Boudreaux et al. (2020) excluded patients under 20 years old. As time passes, a greater insertion rate will translate into a greater fraction of the population using LARC since each device is effective for 3–10 years.

Our results are more consistent with analyses of DelCAN’s effect on LARC insertions in Medicaid which found that DelCAN was as associated with a 10% increase in LARC insertions among adult Medicaid enrollees and a 68% increase among adolescent enrollees [30]. Given that the private reimbursement systems were not reformed as part of the program, the consistency of our results suggests that training, which included clinical and business operations training, and capital support in the form of a “starter” supply of LARC devices, plays a key role in program effects.

To benchmark our findings against other related policies, our estimated effects are similar in magnitude to the ACA contraceptive mandate, which has been estimated as being associated with a 0.85 percentage point increase in the rate of choosing a long term contraceptive method, or a 15% increase from a baseline rate of 5.6 percent [37]. Our results suggest that provider training and public awareness messaging are important complements to improvements in affordability achieved under the ACA.

### Limitations

Our analysis had limitations. As with any difference-in-differences analysis, the underlying assumptions are fundamentally untestable. However, pre-period trends in unadjusted outcome means and our event study model suggest little evidence of pre-trending. We also found that some covariates changed differently in Delaware than the comparison states over time, which suggests some caution in interpreting our results. In particular, we found that the growth in high deductible health plans during the sample period was faster in control states than in Delaware, which may have dampened LARC take-up among enrollees in those states (S4 Table in S1 File). While the ACA mandated in 2011 that all privately insured women have access to LARCs without out-of-pocket costs, participation in HDHPs is known to be associated with reductions in basic preventative care [38]. Reduced health care contacts may have reduced method initiation. However, our models controlled for HDHP enrollment and thus our estimates of interest are unlikely to be substantially affected by changes in HDHP status between Delaware and the comparison states. In total, we failed to reject parallel trends, we come to similar conclusion with and without covariate adjustments, and when using alternative comparison groups and subsamples. The robustness of our results suggest that our results cannot be fully explained by differential compositional shifts. Another limitation is the lack of individual demographic variables available in the Marketscan data. For instance, we do not observe race, income level, educational attainment, employment characteristics, or number of dependents. Marketscan is also a convenience sample and while it is large, its ability to generalize is uncertain. Finally, our study is not able to conclusively speak to how DelCAN affected initiation of other types of contraceptives.

### Conclusions

In this study using a difference-in-differences design, we estimate that a comprehensive statewide initiative including payment reform, clinical training, and a public awareness campaign affected LARC insertion among privately insured women. While this study only identifies the net effect of all the DelCAN interventions on privately insured patients, understanding the effects (and limitations) of such programs is important for informing current and future policymakers in states engaged in similar reforms. Ultimately, DelCAN’s success will be gauged by its impact on unintended pregnancy, if changes in unintended pregnancy were achieved by increasing rather than diminishing patient autonomy, and if the overall cost of the program makes it a cost-effective alternative. However, our results provide important context on the intermediate effects of the program.

## Supporting information

S1 Fig Sample flow diagram. Source: IBM Marketscan Commercial Claims and Encounters Database (2012–2019).
(TIF)

S2 Fig Event study coefficients. Source: IBM Marketscan Commercial Claims and Encounters Database (2012–2019). Notes: Event study estimates are from a linear probability model estimated with individual data, at the person-year level for all LARC insertions and at the birth level for IPP LARC placements. The model controls for state and year fixed effects, individual level covariates and state by year covariates for demographics, health care access, and other state contraceptive policies. See the text for sample inclusion rules and full list of covariates. Standard errors are clustered at the individual level and 95% confidence intervals are shown.
(TIF)

S3 Fig Unadjusted average contraceptive initiation rates among other contraceptive method types. Source: IBM Marketscan Commercial Claims and Encounters Database (2012–2019). Notes: The rate in each panel in each year is calculated as the number of women initiating a method of contraception in that year, divided by the total number of women enrolled in a plan in the Marketscan database. “Any Method” initiation refers to initiation of LARC devices, moderately effective methods, and female sterilization.
(TIF)

S1 File Appendix. Contains all supporting tables.
(DOCX)

S2 File Data availability statement. (DOCX)
