# Supplementary figures and images for: The effect of contraceptive access reform on privately insured patients: Evidence from Delaware Contraceptive Access Now
Source: PLoS One. 2023 Jan 23;18(1):e0280588. doi: 10.1371/journal.pone.0280588 (PMC9870137; doi:10.1371/journal.pone.0280588)

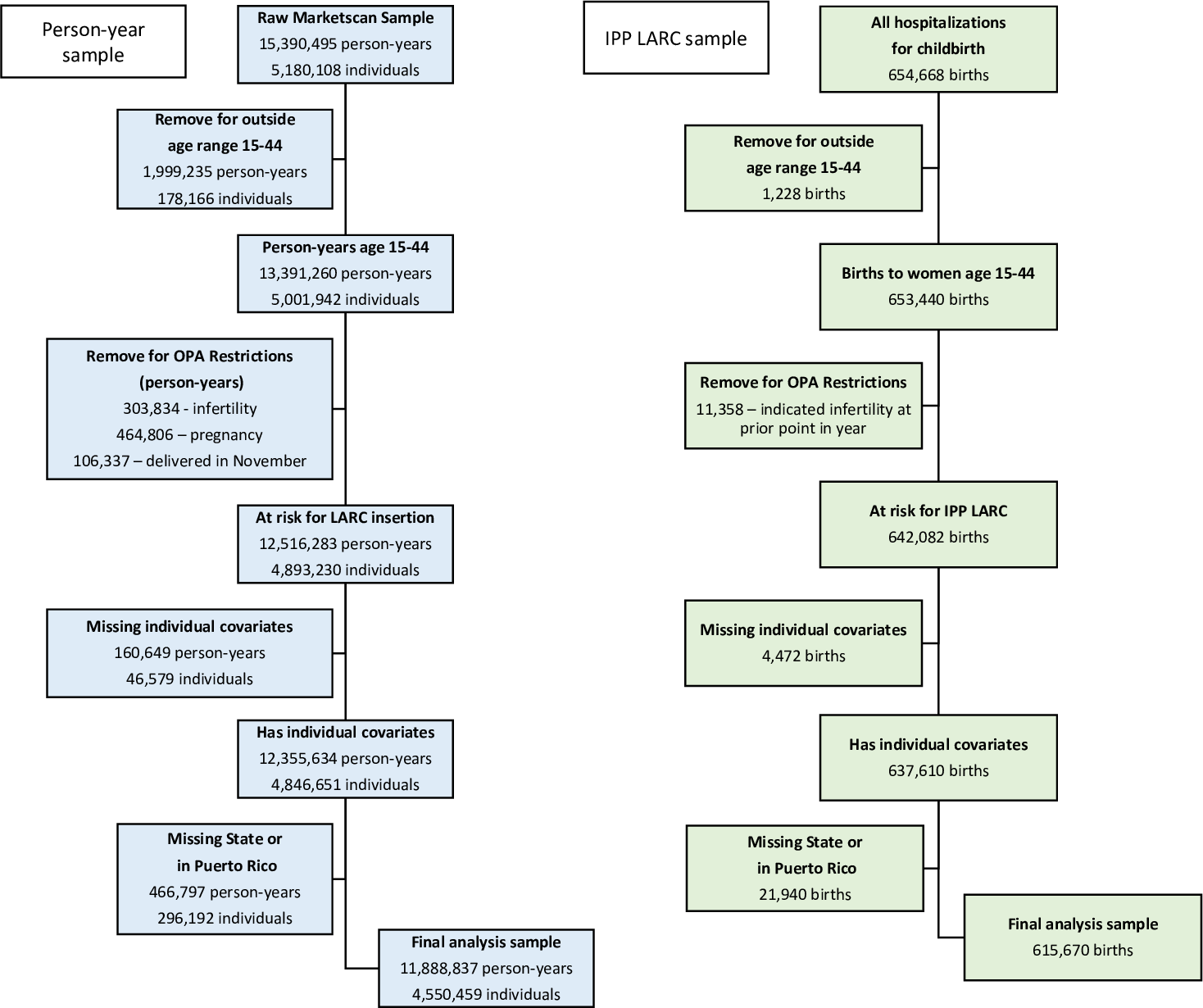

Supplement: S1 Fig — Source: IBM Marketscan Commercial Claims and Encounters Database (2012–2019). (TIF) [file pone.0280588.s001.tif]

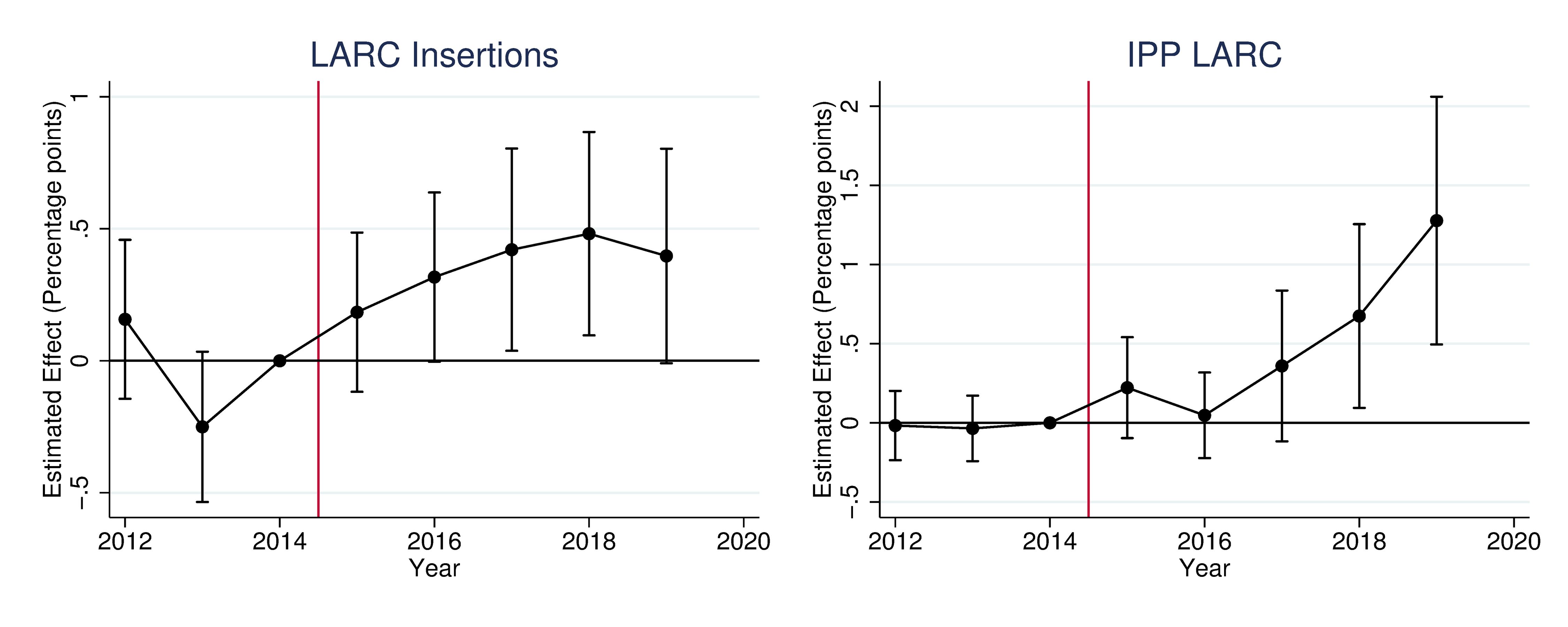

Supplement: S2 Fig — Source: IBM Marketscan Commercial Claims and Encounters Database (2012–2019). Notes: Event study estimates are from a linear probability model estimated with individual data, at the person-year level for all LARC insertions and at the birth level for IPP LARC placements. The model controls for state and year fixed effects, individual level covariates and state by year covariates for demographics, health care access, and other state contraceptive policies. See the text for sample inclusion rules and full list of covariates. Standard errors are clustered at the individual level and 95% confidence intervals are shown. (TIF) [file pone.0280588.s002.tif]

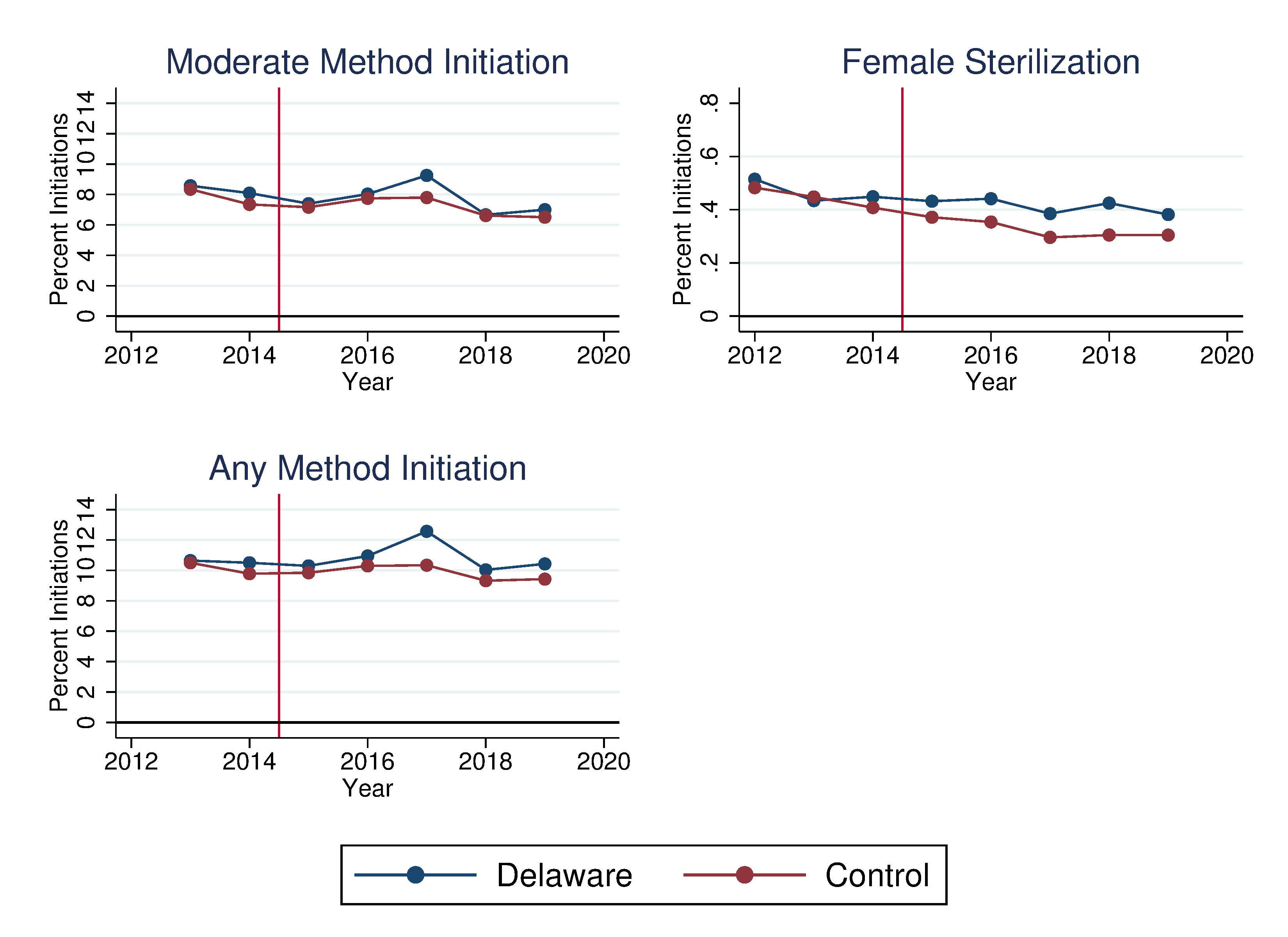

Supplement: S3 Fig — Source: IBM Marketscan Commercial Claims and Encounters Database (2012–2019). Notes: The rate in each panel in each year is calculated as the number of women initiating a method of contraception in that year, divided by the total number of women enrolled in a plan in the Marketscan database. “Any Method” initiation refers to initiation of LARC devices, moderately effective methods, and female sterilization. (TIF) [file pone.0280588.s003.tif]
