# Supplementary material for: The effect of contraceptive access reform on privately insured patients: Evidence from Delaware Contraceptive Access Now
Source: PLoS One. 2023 Jan 23;18(1):e0280588. doi: 10.1371/journal.pone.0280588 (PMC9870137; doi:10.1371/journal.pone.0280588)
Supplement: S1 File — Contains all supporting tables. (DOCX) [file pone.0280588.s004.docx]

**The Effect of Contraceptive Access Reform On Privately Insured Patients: Evidence from Delaware Contraceptive Access Now**

**SUPPORTING INFORMATION**

**Sample Flow Diagram**

Figure S1 presents a diagram of our analytic sample construction. We remove observations from our sample that violate the age restriction, are not at risk for LARC insertion (following guidance from the Office of Population Affairs), or are missing individual-level or state-level covariates.

**Figure S1: Sample Flow Diagram**

**
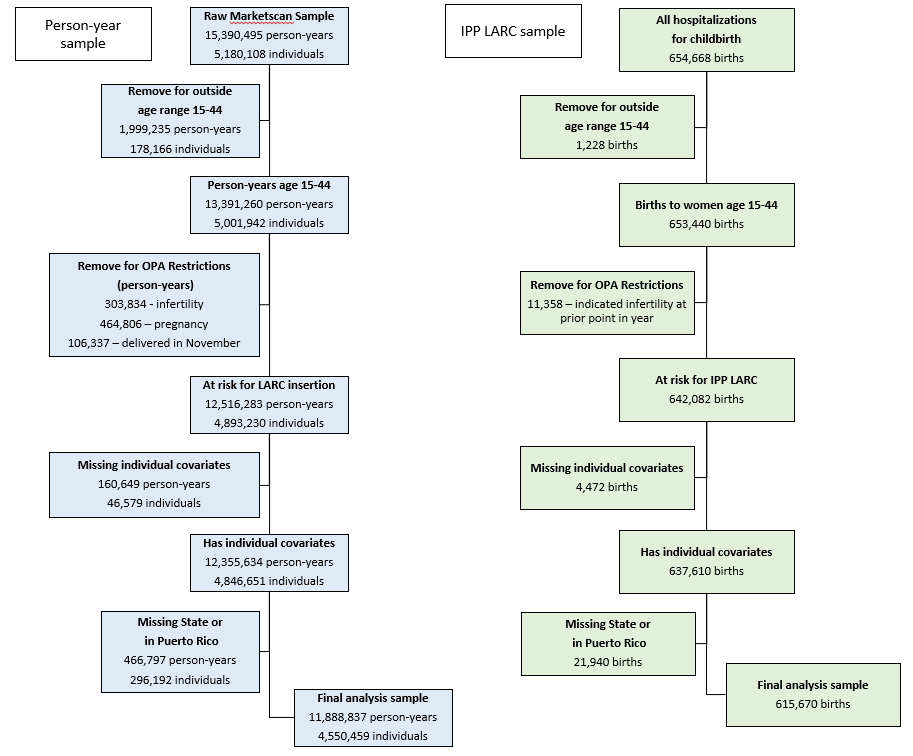
**

Source: IBM Marketscan Commercial Claims and Encounters Database (2012-2019).

**Event Study Analysis**

Figure S2 presents the results of the event study analysis described in the main text. These event study coefficients were obtained from a regression that included all covariates. The results show greater program effects over time for overall LARC insertions and IPP LARC insertions. See Table S2 for further discussion of pre-trends.

**S2 Fig. Event Study Coefficients**


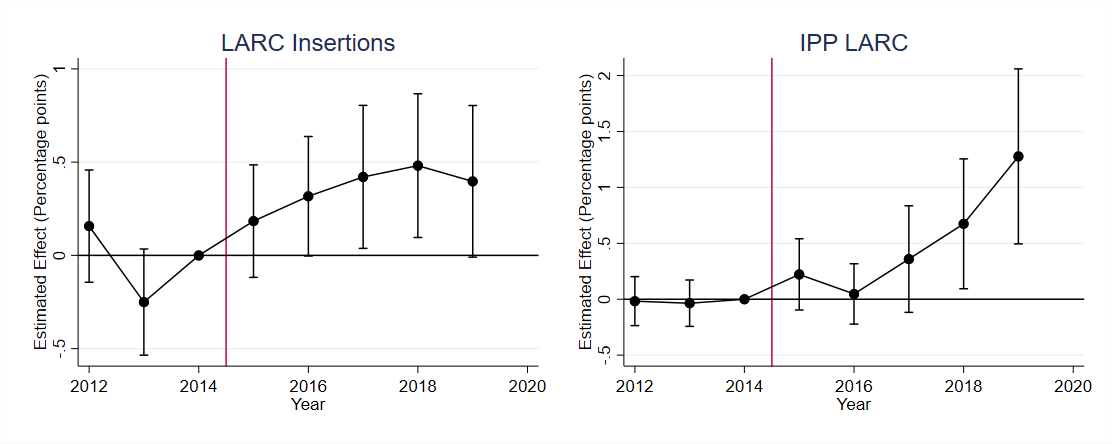


Source: IBM Marketscan Commercial Claims and Encounters Database (2012-2019). Notes: Event study estimates are from a linear probability model estimated with individual data, at the person-year level for all LARC insertions and at the birth level for IPP LARC placements. The model controls for state and year fixed effects, individual level covariates and state by year covariates for demographics, health care access, and other state contraceptive policies. See the text for sample inclusion rules and full list of covariates. Standard errors are clustered at the individual level and 95% confidence intervals are shown.

**Analysis of Alternative Contraceptive Methods**

We analyze the effect of DelCAN on initiation of moderately effective methods (pill, patch, ring, shot, diaphragm) and female sterilization. We also analyze the effect on initiation of any contraceptive method, including LARCs.

Unlike LARCs, moderately effective methods require periodic prescriptions for continued use, so a claim for a moderately effective method does not necessarily indicate a new user. Consistent with previous studies, we identify new moderately effective method users with past claims history.^1^ Specifically, if an enrollee does not have any claims for a moderate method for 6 months preceding a moderate method claim, then we considered them to be a moderately effective method initiator. However, given the trend towards longer prescription fills for some contraceptives and the entry and exit of individuals from the dataset in each year, there is some uncertainty whether this method captures true initiations of moderate methods.

Similar to LARCs, female sterilization is a one-time procedure, so claims for sterilization procedures are considered initiations.

Because moderate method initiation is based on a 6-month look-back, we excluded 2012 from analyses of moderate method initiation because we do not observe claims history prior to 2012. Likewise, we excluded 2012 in analyses of any method initiation. Analyses of LARC and sterilization make use of the full 2012-2019 study period.

Figure S3 presents unadjusted average contraceptive initiation rates of moderately effective methods, sterilization methods, and any contraceptive method.

**S3 Fig. Unadjusted Average Contraceptive Initiation Rates Among Other Contraceptive Method Types**
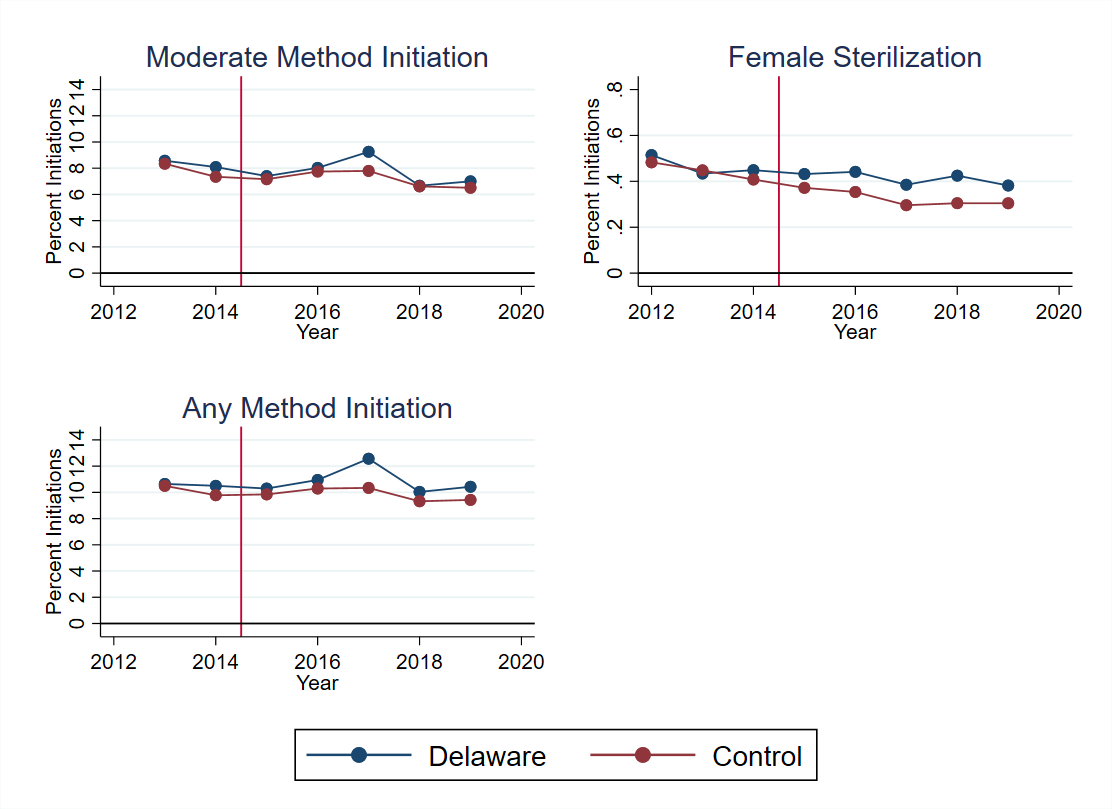


Source: IBM Marketscan Commercial Claims and Encounters Database (2012-2019). Notes: The rate in each panel in each year is calculated as the number of women initiating a method of contraception in that year, divided by the total number of women enrolled in a plan in the Marketscan database. “Any Method” initiation refers to initiation of LARC devices, moderately effective methods, and female sterilization.

We conduct a difference-in-differences analysis similar to the main text. Table S1 presents the results of this analysis. We find that DelCAN was associated with a decline in initiation of moderately effective contraceptive methods of 0.3 percentage points (95% CI [-0.7, 0.1], P=0.12) when adjusting for covariates, representing an 3% decline from Delaware baseline. We failed to find a significant association with female sterilization. Overall, we found initiation of any prescription method rose by 0.2 percentage points (95% CI [-0.2, 0.6], P = 0.37), which is not significant at conventional levels. While our estimates lack precision, the suggestive decline in moderate method use coupled with the modest effect detected for any method initiation suggests that increased LARC insertion most often occurred for enrollees that would have obtained another prescription method in the absence of the program.

However, our analysis of other contraceptive initiations is limited due to potential differential time trends. While the assumptions behind difference-in-differences are fundamentally untestable, we can assess whether the treatment and control group have parallel trends in the outcome variable in the pre-period. Table S2 presents the results of a test of differential pre-trends for LARC methods, moderate methods, sterilization, and any method. See Table S2 for further discussion of pre-trends.

**S1 Table. Difference-in-Differences Estimates For Other Contraceptive Method Types.**

|  | Unadjusted Initiation Rates | | | | Difference-in-Differences | |
| --- | --- | --- | --- | --- | --- | --- |
|  | Delaware | | Control | |  |  |
|  | Pre | Post | Pre | Post | Unadjusted | Adjusted |
| Moderate Methods | 12.0 | 11.0 | 11.6 | 11.2 | -0.4** [-0.7, -0.1] | -0.3 [-0.7, 0.1] |
| Female Sterilization | 0.6 | 0.6 | 0.6 | 0.5 | 0.1† [0.0, 0.1] | 0.0 [0.0, 0.1] |
| Any Method | 15.2 | 15.6 | 14.9 | 15.2 | 0.1 [-0.2, 0.5] | 0.2 [-0.2, 0.6] |

Source: IBM Marketscan Commercial Claims and Encounters Database (2012-2019). Notes: All estimates are in percentage points. Both the unadjusted and adjusted difference-in-difference estimates include state and year fixed effects. The adjusted model controls for individual level covariates and state by year covariates for demographics, health care access, and other state contraceptive policies. See the text for sample inclusion rules and full list of covariates. Statistical tests account for clustered standard errors at the individual level and 95% confidence intervals are shown. †p<0.1, *p<0.05; **p<0.01; ***p<0.001.

**Differential Pre-Trends Analysis**

Table S2 describes the p-value on a test of differential pre-period trends. The test allows us to examine the plausibility of the parallel trends assumption which underlies our difference-in-differences design. This test was conducted by regressing the outcome on year, a Delaware indicator, and the interaction of year and the Delaware indicator, using only pre-period data. For the LARC and sterilization outcomes, the coefficient of interest is on the interaction of continuous year and the Delaware indicator, which shows whether Delaware had a different slope over time in the pre-period compared to other states.

In the LARC and sterilization models, the p-value on the interaction term was large and we cannot reject the equality of pre-period trends in Delaware and the comparison states. This suggests that the parallel trends assumption holds. For the Moderate and Any Method outcomes, the p-value suggested differences in trend that were statistically significant which diminishes our confidence. However, the Moderate Method and Any Method outcomes have only 2 pre-period years, as discussed previously. As a result, the coefficient on the interaction of year and the Delaware indicator represents differential change from 2013 to 2014 in Delaware compared to the comparison states. Thus, the pre-period may be too short to detect any meaningful trends in these outcomes.

**S2 Table. Pre-Trends Test**

|  | Linear Trends p-value |
| --- | --- |
| LARC | 0.599 |
| IPP LARC | 0.729 |
|  |  |
| Moderate Methods | 0.005 |
| Female Sterilization | 0.989 |
| Any Contraceptive Method | 0.005 |

Source: IBM Marketscan Commercial Claims and Encounters Database (2012-2019). Notes: Estimates are from a linear probability model estimated with individual data, at the person-year level. The model does not control for any additional covariates not mentioned above. See the text for sample inclusion rules. Standard errors are clustered at the individual level.

**Alternative Control Groups Analysis**

We analyze the sensitivity of our results to a number of alternative control groups. Our baseline control group uses individuals from 43 US states and the District of Columbia. We already remove individuals from six US states (Colorado, Iowa, Massachusetts, South Carolina, Texas, and Washington) due to particularly active contraceptive policy reform in those states. Our baseline assumption is that absent the program, Delaware would have a trajectory with contraceptive access reform similar to the average US state. However, we show our results are robust to several other choices of a comparison group.

We construct two alternative control groups based on additional contraceptive policy features. The contraceptive policies we considered included whether the state mandated insurance coverage of over-the-counter contraceptives, male vasectomy, or an extended supply of birth control pills in a single prescription. We also considered whether pharmacists in the state were permitted to prescribe contraceptives and whether the state Medicaid program rules reimbursed separately for IPP LARC placements. For reference, Delaware mandated insurance coverage of over-the-counter contraceptives and extended supplies of birth control pills in 2018, and implemented Medicaid IPP LARC payment carve-out in 2015 as part of DelCAN, but did not adopt the other contraceptive access policies during the study period.

The first alternative control group removes additional states that were relatively active regarding these contraceptive policies. Specifically, this group removes individuals from California, Maryland, New Mexico, Oregon, and the District of Columbia. These states or territories mandated coverage for extended supply of birth control pills and allowed greater pharmacist prescribing power for several years during the study period, and had Medicaid reimburse separately for IPP LARC starting at or before 2015. These states have the most widespread adoption of the contraceptive policies considered.

We also construct a control group of states that had the most similar profile to Delaware in terms of contraceptive policy. This control group consists of individuals from Connecticut, Idaho, Illinois, Indiana, and Montana. Like Delaware, these states implemented separate Medicaid reimbursement for IPP LARC in 2015, and adopted one or two of the other contraceptive policies for a few years during the study period.

Finally, we consider control groups of neighboring states. First, we consider the close neighboring states of Maryland, Virginia, Pennsylvania, and New Jersey. We also augment this control group by adding individuals from the District of Columbia and West Virginia. For these specifications, we remove the policy controls for mandated insurance coverage of over-the-counter contraceptives and extended supplies of birth control pills from the adjusted model. This adjustment is due to Delaware having passed both these policies in 2018 and the neighboring states largely did not. As a result, these policy covariates are highly collinear with the treatment.

Table S3 presents the results of the difference-in-differences analysis for each of these control groups.

**S3 Table. Difference-in-Differences Estimates For Alternative Control Groups**

|  | Unadjusted Initiation Rates | | | | Difference-in-Differences | |
| --- | --- | --- | --- | --- | --- | --- |
|  | Delaware | | Control | |  |  |
|  | Pre | Post | Pre | Post | Unadjusted | Adjusted |
| **All LARC Initiations** |  |  |  |  |  |  |
| Baseline | 3.0 | 4.3 | 3.1 | 4.0 | 0.5*** [0.3, 0.6] | 0.3*** [0.2, 0.5] |
| Remove Most Active States | 3.0 | 4.3 | 3.1 | 4.0 | 0.5*** [0.3, 0.6] | 0.4*** [0.2, 0.6] |
| Most Similar to DE | 3.0 | 4.3 | 3.2 | 4.1 | 0.4*** [0.2, 0.6] | 1.0*** [0.6, 1.3] |
| Neighboring States‡ | 3.0 | 4.3 | 2.5 | 3.6 | 0.3*** [0.1, 0.5] | 0.3† [0.0, 0.6] |
| Neighboring States +DC,WV‡ | 3.0 | 4.3 | 2.5 | 3.7 | 0.3*** [0.1, 0.5] | 0.2 [-0.1, 0.4] |
|  |  |  |  |  |  |  |
| **IPP LARC Initiations** |  |  |  |  |  |  |
| Baseline | 0.1 | 0.6 | 0.1 | 0.2 | 0.4*** [0.2, 0.6] | 0.4*** [0.2, 0.6] |
| Remove Most Active States | 0.1 | 0.6 | 0.1 | 0.2 | 0.4*** [0.2, 0.6] | 0.4*** [0.2, 0.6] |
| Most Similar to DE | 0.1 | 0.6 | 0.1 | 0.2 | 0.5*** [0.3, 0.7] | 0.6*** [0.3, 0.9] |
| Neighboring States‡ | 0.1 | 0.6 | 0.1 | 0.2 | 0.4*** [0.2, 0.7] | 0.5* [0.1, 0.9] |
| Neighboring States +DC,WV‡ | 0.1 | 0.6 | 0.1 | 0.2 | 0.4*** [0.2, 0.7] | 0.4** [0.1, 0.8] |

Source: IBM Marketscan Commercial Claims and Encounters Database (2012-2019). Notes: All estimates are in percentage points. Both the unadjusted and adjusted difference-in-difference estimates include state and year fixed effects. The adjusted model controls for individual level covariates and state by year covariates for demographics, health care access, and other state contraceptive policies. See the text for sample inclusion rules and full list of covariates. Statistical tests account for clustered standard errors at the individual level and 95% confidence intervals are shown. †p<0.1, *p<0.05; **p<0.01; ***p<0.001.

‡ We remove control variables in the adjusted model for state mandated insurance coverage of over-the-counter contraceptives and extended supply of birth control pills for these specifications because these variables are highly collinear with the treatment.

**Covariate Balance Analysis**

We analyze the covariate balance of our analysis samples to understand if there are changes in the composition of our sample over time that could bias our results. Table S4 shows means of observed characteristics by year and treatment group. Table S5 formally tests for differences in covariate balance using a regression-based test. Specifically, the test regresses each covariate on the interaction of the Delaware indicator and the post period indicator, with state and year fixed effects. The coefficient of interest is on the interaction of the Delaware indicator and post period indicator, which shows whether observations in Delaware were more likely to have different values of the covariates in the post period relative to other states.

Together, these results show that our sample does have some shifts in observable characteristics over time. The groups are balanced on age composition, and enrollees in Delaware are 2-3 percentage points more likely to be policyholders in the post period compared to the control group. While both treatment and control groups trend towards greater enrollment in HDHP plans, this trend is greater in the control group. Most striking, the share of the sample that is urban increases by 8 percentage points in Delaware, net of the change observed in the control group (Table S5).

While our main models control for urban status, we also investigated if our results were robust in the subsample of cases living in urban areas. Our main results reported in Table 2 are similar when only using the subsample of enrollees living in urban areas (Table S6). However, the urban subsample is still similarly unbalanced regarding HDHP and policyholder status (Table S7).

Differential changes in sample composition are a source of concern. However, our results are robust with and without the inclusion of covariates, in the subsample of urban patients, using alternative control groups, and we fail to reject parallel trends. These sensitivity results suggest that our preferred estimates in Table 2 (main text) are unlikely to be explained by compositional shifts. Another natural check of this assertion would be to limit the sample to a longitudinal panel of patients that we observe in every year. However, imposing that constraint would retain less than 5% of the sample and would introduce new compositional concerns due to cohort aging and pregnancy.

**S4 Table. Summary Statistics by Year and Treatment Group**

|  | **2012** | **2013** | **2014** | **2015** | **2016** | **2017** | **2018** | **2019** |
| --- | --- | --- | --- | --- | --- | --- | --- | --- |
| **Person-Year Sample** | | | | | | | | |
| ***Delaware*** |  |  |  |  |  |  |  |  |
| Age (years) | 29.6 | 29.5 | 29.4 | 29.3 | 29.6 | 29.8 | 29.7 | 29.8 |
| Urban (%) | 83.7 | 84.7 | 100 | 100 | 100 | 100 | 100 | 100 |
| Policyholder (%) | 48.7 | 48.1 | 47.6 | 48.5 | 48.6 | 50 | 49.1 | 48.8 |
| HDHP (%) | 1.3 | 2.2 | 2.7 | 2.4 | 2.9 | 4.0 | 4.2 | 5.3 |
| Postpartum (%) | 4.5 | 4.2 | 4.5 | 4.6 | 4.5 | 4.3 | 4.2 | 4.2 |
| N | 28,768 | 29,995 | 30,886 | 32,630 | 33,466 | 18,390 | 27,371 | 22,407 |
|  |  |  |  |  |  |  |  |  |
| ***Control States*** | | | | | | | | |
| Age (years) | 29.6 | 29.7 | 29.6 | 29.4 | 29.9 | 29.9 | 29.8 | 29.9 |
| Urban (%) | 85.0 | 86.1 | 86.8 | 88.2 | 88.5 | 90.0 | 90.1 | 90.4 |
| Policyholder (%) | 44.9 | 45.0 | 44.9 | 44.7 | 45.2 | 45.0 | 44.2 | 42.8 |
| HDHP (%) | 3.8 | 6.0 | 7.4 | 7.6 | 8.8 | 11.3 | 13.0 | 14.5 |
| Postpartum (%) | 4.6 | 4.5 | 4.6 | 4.6 | 4.5 | 4.5 | 4.4 | 4.4 |
| N | 1,899,249 | 1,759,769 | 1,745,909 | 1,470,811 | 1,458,521 | 1,196,409 | 1,153,018 | 981,238 |
|  |  |  |  |  |  |  |  |  |
| **IPP LARC Sample** | | | | | | | | |
| ***Delaware*** |  |  |  |  |  |  |  |  |
| Age (years) | 30.2 | 30.1 | 30.3 | 30.1 | 30.1 | 30.5 | 30.7 | 30.7 |
| Urban (%) | 81.4 | 85.0 | 100 | 100 | 100 | 100 | 100 | 100 |
| Policyholder (%) | 54.4 | 53.7 | 56.0 | 55.3 | 56.2 | 58.1 | 57.1 | 61.2 |
| HDHP (%) | 0.9 | 2.2 | 2.2 | 1.8 | 2.0 | 3.5 | 2.6 | 5.4 |
| N | 1,477 | 1,427 | 1,586 | 1,689 | 1,690 | 887 | 1,295 | 1,082 |
|  |  |  |  |  |  |  |  |  |
| ***Control States*** | | | | | | | | |
| Age (years) | 30.1 | 30.2 | 30.3 | 30.4 | 30.4 | 30.5 | 30.8 | 30.8 |
| Urban (%) | 85.3 | 86.6 | 87.0 | 88.5 | 88.3 | 89.7 | 89.6 | 89.8 |
| Policyholder (%) | 47.9 | 48.8 | 49.4 | 48.7 | 49.4 | 48.7 | 48.4 | 48.1 |
| HDHP (%) | 3.7 | 5.8 | 7.7 | 7.8 | 9.1 | 11.7 | 13.3 | 14.9 |
| N | 101,049 | 91,290 | 91,219 | 77,334 | 75,142 | 60,935 | 58,118 | 49,450 |

Source: IBM Marketscan Commercial Claims and Encounters Database (2012-2019). Notes: Observations for all LARC initiations are at the “person-year” level and observations for IPP LARC are at the birth level. “Urban” refers to the percentage of person-year observations where the enrollee was located in a Metropolitan Statistical Area.

**S5 Table. Covariate Balance Test**

| **Variable** | **Estimate** | **95% Confidence Interval** |
| --- | --- | --- |
| **Person-Year Sample** |  |  |
| Age (years) | 0.1 | [0.0, 0.2] |
| In urban area (%) | 8.0*** | [7.7, 8.2] |
| Policyholder (%) | 1.5*** | [1.0, 2.0] |
| High deductible health plan (%) | -3.4*** | [-3.6, -3.2] |
| Postpartum (%) | 0.1 | [-0.1, 0.3] |
|  |  |  |
| **Postpartum Sample** |  |  |
| Age (years) | -0.1 | [-0.4, 0.1] |
| In urban area (%) | 8.9*** | [8.0, 9.8] |
| Policyholder (%) | 2.6** | [0.8, 4.5] |
| High deductible health plan (%) | -4.2*** | [-4.8, -3.6] |

Source: IBM Marketscan Commercial Claims and Encounters Database (2012-2019). Notes: Estimates are from a linear probability model estimated with individual data, where the outcome is the listed covariate. The model includes state and year fixed effects but does not control for any additional covariates. Standard errors are clustered at the individual level. †p<0.1, *p<0.05; **p<0.01; ***p<0.001.

**S6 Table. Difference-in-Differences Estimates for Urban Enrollees**

|  | Unadjusted Initiation Rates | | | | Difference-in-Differences | |
| --- | --- | --- | --- | --- | --- | --- |
|  | Delaware | | Control | |  |  |
|  | Pre | Post | Pre | Post | Unadjusted | Adjusted |
| All LARC Initiations | 3.1 | 4.3 | 3.1 | 4.0 | 0.3*** [0.2, 0.5] | 0.2† [0.0, 0.4] |
| IPP LARC Initiations | 0.1 | 0.6 | 0.1 | 0.3 | 0.4*** [0.2, 0.6] | 0.4*** [0.2, 0.6] |

Source: IBM Marketscan Commercial Claims and Encounters Database (2012-2019). Notes: Only includes enrollees identified as living in a Metropolitan Standard Area. All estimates are in percentage points. Both the unadjusted and adjusted difference-in-difference estimates include state and year fixed effects. The adjusted model controls for individual level covariates and state by year covariates for demographics, health care access, and other state contraceptive policies. See the text for sample inclusion rules and full list of covariates. Statistical tests account for clustered standard errors at the individual level and 95% confidence intervals are shown. †p<0.1, *p<0.05; **p<0.01; ***p<0.001.

**S7 Table. Covariate Balance Test for Urban Enrollees**

| **Variable** | **Estimate** | **95% Confidence Interval** |
| --- | --- | --- |
| **Person-Year Sample** |  |  |
| Age (years) | 0.0 | [-.08, 0.10] |
| In urban area (%) | 0.0 | [0.0, 0.0] |
| Policyholder (%) | 0.9*** | [0.4, 1.4] |
| High deductible health plan (%) | -3.6*** | [-3.8, -3.5] |
| Postpartum (%) | 0.2 | [0.0, 0.3] |
|  |  |  |
| **Postpartum Sample** |  |  |
| Age (years) | -0.3** | [-.51, -.09] |
| In urban area (%) | 0.0 | [0.0, 0.0] |
| Policyholder (%) | 1.9† | [-0.1, 3.9] |
| High deductible health plan (%) | -4.4*** | [-5.0, -3.8] |

Source: IBM Marketscan Commercial Claims and Encounters Database (2012-2019). Notes: Only includes enrollees identified as living in a Metropolitan Standard Area. Estimates are from a linear probability model estimated with individual data, where the outcome is the listed covariate. The model includes state and year fixed effects but does not control for any additional covariates. Standard errors are clustered at the individual level. †p<0.1, *p<0.05; **p<0.01; ***p<0.001.

**Baseline Characteristics by Age Group**

In this section, we explore differences in reported characteristics by age group to further understand the greater response to the program among adolescents. Table S8 presents outcome and covariate means by age group, treatment group, and time period. While adolescents live in urban areas and are enrolled in high deductible health plans at similar rates as adults, they are less likely to be policyholders or be in the postpartum period. Trends over time and across treatment and control group are similar for teenage and adult enrollees. Both being a policyholder and being postpartum are positively associated with LARC initiation, so this difference may explain the lower total rates of LARC initiation among enrollees age 15-17 versus age 18-29. However, we estimate the response to the program is greater for adolescent enrollees, both in absolute and percentage terms.

**S8 Table. Summary Statistics by Age Group**

|  | **Delaware** | | **Control States** | |
| --- | --- | --- | --- | --- |
|  | **Pre-Period** | **Post-Period** | **Pre-Period** | **Post-Period** |
| **Person-Year Sample** | | | | |
| **Age 15-17:** |  |  |  |  |
| LARC Initiation (%) | 2.2 | 3.6 | 1.9 | 2.3 |
| Age (years) | 16.0 | 16.0 | 16.0 | 16.0 |
| Urban (%) | 88.4 | 100.0 | 84.8 | 88.1 |
| Policyholder (%) | 0.4 | 0.5 | 0.9 | 0.2 |
| HDHP (%) | 1.9 | 3.7 | 5.7 | 11.4 |
| Postpartum (%) | 0.3 | 0.3 | 0.4 | 0.2 |
| N | 9,624 | 12,446 | 540,875 | 560,905 |
| **Age 18-29:** |  |  |  |  |
| LARC Initiation (%) | 3.7 | 5.5 | 4.0 | 5.1 |
| Age (years) | 23.0 | 23.2 | 23.2 | 23.3 |
| Urban (%) | 89.4 | 100.0 | 86.0 | 89.1 |
| Policyholder (%) | 33.4 | 30.8 | 34.2 | 31.5 |
| HDHP (%) | 1.9 | 3.2 | 5.5 | 10.2 |
| Postpartum (%) | 5.0 | 4.4 | 5.4 | 4.6 |
| N | 34,178 | 53,819 | 2,063,827 | 2,484,722 |
| **Age 30-44:** |  |  |  |  |
| LARC Initiation (%) | 2.7 | 3.6 | 2.7 | 3.4 |
| Age (years) | 37.1 | 37.2 | 37.0 | 37.1 |
| Urban (%) | 90.1 | 100.0 | 86.1 | 89.6 |
| Policyholder (%) | 69.1 | 72.1 | 61.4 | 62.2 |
| HDHP (%) | 2.3 | 3.9 | 5.9 | 10.9 |
| Postpartum (%) | 4.8 | 5.1 | 4.7 | 5.2 |
| N | 45,847 | 67,999 | 2,800,225 | 3,214,370 |
| **IPP LARC Sample** | | | | |
| **Age 15-29:** |  |  |  |  |
| IPP Initiation (%) | 0.0 | 1.2 | 0.1 | 0.3 |
| Age (years) | 25.0 | 25.4 | 25.3 | 25.4 |
| Urban (%) | 86.6 | 100.0 | 81.2 | 83.8 |
| Policyholder (%) | 42.5 | 45.2 | 40.8 | 39.6 |
| HDHP (%) | 1.4 | 2.3 | 5.2 | 10.0 |
| N | 1,863 | 2,726 | 121,766 | 128,947 |
| **Age 30-44:** |  |  |  |  |
| IPP Initiation (%) | 0.1 | 0.3 | 0.1 | 0.2 |
| Age (years) | 33.8 | 33.8 | 33.9 | 34.0 |
| Urban (%) | 90.9 | 100.0 | 90.1 | 92.6 |
| Policyholder (%) | 63.5 | 65.5 | 54.6 | 54.8 |
| HDHP (%) | 2.1 | 3.2 | 6.0 | 11.6 |
| N | 2,627 | 3,917 | 161,792 | 192,032 |

Source: IBM Marketscan Commercial Claims and Encounters Database (2012-2019). Notes: Observations for all LARC initiations are at the “person-year” level and observations for IPP LARC are at the birth level. “Urban” refers to the percentage of person-year observations where the enrollee was located in a Metropolitan Statistical Area.

**References**

1. Pace LE, Dusetzina SB, Keating NL. Early Impact Of The Affordable Care Act On Oral Contraceptive Cost Sharing, Discontinuation, And Nonadherence. *Health Aff (Millwood)*. 2016;35(9):1616-1624. doi:10.1377/hlthaff.2015.1624
