# Supplementary material for: The effect of contraceptive access reform on privately insured patients: Evidence from Delaware Contraceptive Access Now
Source: PLoS One. 2023 Jan 23;18(1):e0280588. doi: 10.1371/journal.pone.0280588 (PMC9870137; doi:10.1371/journal.pone.0280588)
Supplement: S2 File — (DOCX) [file pone.0280588.s005.docx]

**The Effect of Contraceptive Access Reform On Privately Insured Patients: Evidence from Delaware Contraceptive Access Now**

**DATA AVAILABILITY STATEMENT**

Health insurance claims from employer sponsored insurance were obtained from the IBM Marketscan Commercial Claims and Encounters Database. The Data Use Agreement with IBM Marketscan prohibits data sharing and distribution. This data source provides enrollment information and inpatient, outpatient, and prescription drug claims of individuals and families enrolled in employer sponsored insurance nationwide. Since the initiation of our DUA with IBM, the Marketscan Database was acquired by Merative.

To obtain access to the Marketscan database, please visit <https://www.ibm.com/products/marketscan-research-databases/databases>.

The base code sets and algorithms for identifying at-risk patient cohorts and their contraceptive method types were based on publicly available guidance and SAS coding from the Office of Population Affairs, available at <https://opa.hhs.gov/research-evaluation/title-x-services-research/contraceptive-care-measures>

We obtained a set of state-by-year covariates from the publicly available American Community Survey and the Area Health Resource File. We also include a set of indicators for state laws on a number of reproductive health policies. These policies are collected by the study team.^[[1]](#footnote-1)^ These covariates are available for download on Github, at the following repository:

<https://github.com/maranna-yoder/delcan-esi>

1. For a fuller description of these data, please see https://udspace.udel.edu/handle/19716/29076. [↑](#footnote-ref-1)
